# Students prefer personalized, AI-generated educational videos over non-personalized, human-recorded videos

**DOI:** 10.1038/s41598-026-52798-9

**Published:** 2026-05-13

**Authors:** Bill Tomlinson, Rebecca W. Black, Donald J. Patterson, André van der Hoek, Julie Ferguson, Matthew J. Bietz

**Affiliations:** 1 Department of Informatics, Donald Bren School of Information and Computer Sciences, University of California, Irvine, CA USA; 2 Department of Earth System Science, University of California, Irvine, CA USA

**Keywords:** Personalized learning, Generative AI, Educational video, Online education, Student preference, Field deployment, Mathematics and computing, Psychology, Psychology, Science, technology and society

## Abstract

Personalization is a well-established driver of student engagement, yet delivering individualized instruction at scale remains a challenge in online education. Recent advances in generative AI make scalable personalization feasible, but AI-generated educational videos are often perceived as inferior to human-recorded content. This tension raises the question: how does the value of personalization compare to that of human presence? We investigated this question through a field deployment in two offerings of a large undergraduate online course (493 respondents). AI-generated personalized videos served as the primary instructional modality, alongside a smaller set of non-personalized human-recorded and AI-generated videos. At the end of the course, students ranked preferences across personalized and non-personalized formats and human-recorded versus AI-generated content. In a direct comparison, students preferred AI-generated personalized videos over human-recorded non-personalized videos (mean rank 2.26 vs. 2.69; Wilcoxon signed-rank test, $p < 0.001$). Across analyses, students preferred personalized over non-personalized content, and human-recorded over AI-generated content. The magnitude of the personalization effect substantially exceeded the effect of human presence. Open-ended responses highlighted perceived benefits of relevance and conciseness in personalized AI videos, alongside concerns about naturalness and expressiveness. Together, these findings suggest that personalization can outweigh human presence in students’ evaluations of educational video.

## Introduction

Video-based instruction has become an important component of many contemporary higher education contexts, particularly in online and hybrid learning environments^1–3^. Educational videos offer flexibility, scalability, and consistency, but they are typically produced as one-size-fits-all resources, presenting identical content to all learners. This uniformity limits their ability to address variation in students’ interests, backgrounds, and prior knowledge—an issue long emphasized in the learning sciences literature as a limitation of lecture-centered instructional approaches^4^.

Personalized instruction, by contrast, is a well-established driver of student engagement and learning. Classic work on individualized tutoring demonstrated that one-to-one instruction can yield learning gains of up to two standard deviations relative to conventional classroom teaching, a phenomenon widely known as the “two sigma problem”^5^. Subsequent research has shown that even partial forms of instructional personalization—such as adapting pacing and instructional support to learners’ needs—can improve academic performance and student attitudes toward instruction^6^. Despite these benefits, personalization has historically been difficult to implement at scale, particularly in large courses, due to the substantial time and labor required of instructors.

Recent advances in generative artificial intelligence (AI) have renewed interest in scalable personalization^7^. Large language models can rapidly produce instructional content and support personalized learning experiences^8^.. Empirical evidence from authentic educational settings suggests that AI-driven tutoring and instructional systems can be effective. In one field study, an AI-based virtual tutor achieved learning outcomes that exceeded those of in-class active learning interventions^9^. In another study, research shows that students use AI systems as effective learning supports in large undergraduate courses, aiding understanding, research, and writing while maintaining intellectual independence^10^.

Prior research comparing human-recorded and AI-generated educational videos has reported mixed results, often revealing a tension between learner experience and objective learning outcomes. In particular, experimental work comparing human-recorded and AI-generated teaching videos suggests a tradeoff: while human-made videos tend to confer a statistically significant but modest advantage in perceived learning experience, objective learning outcomes are often comparable across human and AI-generated instruction^11^. These differences are commonly attributed to social and attentional cues in instruction, including eye gaze and on-screen presence, which human instructors use to signal focus and foster a sense of interpersonal interaction^12^. AI-generated presenters may also elicit “uncanny valley” effects, in which near-human appearance or behavior evokes discomfort or distrust^13^.

Taken together, prior work highlights a tension between two influential factors in educational video design: personalization and human presence. Personalization increases relevance and engagement, while human-delivered instruction provides social cues and affective qualities that many learners value. Existing studies rarely disentangle these dimensions, often comparing human and AI instruction without personalization, or examining personalization within a single delivery modality. As a result, it remains unclear how students weigh personalization against human presence when evaluating educational video content.

This study is based on the premise that perceptions of technology are an important factor in acceptance of the technology within a specific context, a premise that underlies a good deal of research on information systems. For example, the Technology Acceptance Model (TAM) posits that intentions to use a technology lead to actual use of the technology^14,15^. While the Technology Acceptance Model was developed within a business context, its value has been demonstrated within educational contexts as well^16,17^. While the TAM focuses on usability and usefulness, here we explore two additional factors that likely affect intention to use for AI-based technology in pedagogical settings.

The present study addresses this gap by examining how students evaluate educational videos within a preference space defined by two orthogonal dimensions: personalization (personalized versus non-personalized) and source (human-recorded versus AI-generated). We report results from a field deployment in a large undergraduate online course in which AI-generated, interest-personalized videos served as the primary instructional modality throughout the term. In addition to these personalized AI videos, the course included non-personalized human-recorded lecture videos intended to complement the AI content, as well as a small number of non-personalized AI-generated videos, giving students exposure to multiple instructional formats during the term. Many students in the course population had prior experience with online and video-based learning, as indicated by surveys from recent offerings of the same course. At course end, students ranked their preferences among four video types representing these dimensions, drawing on a combination of direct course experience, prior exposure to online instruction, and informed hypothetical comparison. By analyzing both quantitative rankings and students’ qualitative explanations, this study seeks to clarify whether the benefits of personalization can outweigh students’ preference for human presence in video-based instruction.

### AI-generated personalized videos

This study was based on a set of videos produced for a large online undergraduate course taught at a major U.S. research university. The course focused on connections between information technology and global sustainability.

The team built a custom software pipeline to maximally benefit from AI support while still allowing human oversight and contributions at multiple stages of production. Before we generated any videos, the instructor of the course iteratively developed an LLM prompt that represented his “ethos”, reflecting his broad view of the set of topics being discussed in the course as well as his pedagogical style and authorial voice. The instructor also worked with AI to select 100 topics to be covered in the course. For each of these topics (e.g., “Sustainability”, “Feedback Loops”), the team generated three versions of the video, one tailored to business, one tailored to technology, and one tailored to society/biology. These three topics were chosen via an AI-based analysis of student majors in the first of the two course offerings studied here, with the goal of selecting a set of three topics that would most effectively appeal to the student population.

Each video was based on a 5-paragraph script. The three versions of each video were identical for the first three paragraphs, which presented the core content of the topic; in the last two paragraphs, they diverged to present examples connecting the topic to one of the three specific domains.

To generate the first three paragraphs for each set of scripts, the team prompted an LLM (OpenAI’s LLM GPT-4o^18^ or Anthropic’s Claude 3.7 Sonnet^19^) with the instructor’s “ethos”, the topic of the video, and the structure of those three paragraphs. The prompt also included any “hints” that the instructor wanted to be included, such as specific examples or data to be included. Finally, the prompt also included a list of terms to avoid such as “ever-changing”, “tapestry”, “truly”, and “unlock the secrets”, and idioms to avoid such as the inclusion of a call to action at the end of a speech act.

The system then generated three sets of the final two paragraphs. The prompt was similar to the prompt for the first two paragraphs, but tailored to the specific interests of one of the three groups.

The instructor and project scientist then read all the scripts, and edited them as needed. Sometimes the script was discarded and re-generated, either with the same or a different LLM. All examples were checked by the instructor for correctness. This was a time-consuming process, but still quicker than it would have been to write multiple versions of 100 scripts by hand.

The scripts were then returned to the pipeline to have them made into videos. The videos involved compositing and editing together an AI avatar, bullet point summaries, images, titles, credits, and music.

For the AI avatar, the team used the HeyGen system^20^. The avatar was produced based on a recording of the instructor. It took numerous iterations to adjust the expressiveness, intonation, lighting, and framing of the source recording to produce a satisfactory avatar. We recorded avatars in three configurations: straight-ahead, left-facing, and right-facing, so that we could generate more diverse and engaging videos. From these avatars, we rendered each paragraph of each script as an AI-generated video.

The images were primarily selected from the Unsplash^21^ photo library. The team built a system to retrieve an appropriate image, based on a set of search terms derived from that paragraph of the script. The team then reviewed each image for appropriateness and replaced any that were not deemed suitable for the content. Credits for each image were collected for inclusion in the credits.

Another LLM call was used to convert each paragraph of the script into a set of bullet points; one bullet point per sentence. These were also reviewed by the team for correctness.

The avatar videos, images, and bullet points where then dynamically composited with titles, credits, and music into a final video form. The total length of each video ranged from approximately 3 to 5 minutes. The final videos were reviewed for correctness.

Finally, the team’s software pipeline uploaded the video to Youtube with appropriate metadata. From each set of three Youtube videos, a single page of content was dynamically created on the Canvas learning management system. A total of 100 pages were created, each with three videos on them.

Students accessed the material through Canvas, where they were given the option to view any one of the three versions of the videos about each topic. They were shown approximately 10 topics per week, across 10 weeks.

In addition to these videos, the course content included several non-personalized AI videos (one introducing the AI avatar and the technology being used to create the videos, and several end-of-week summaries tying together the course material). There were also 25 human-recorded, non-personalized videos of various types: course introduction and conclusion, weekly anecdotes, field trips, interviews with experts, and teaching staff meeting updates. These videos were intended to be complementary to the core content, which was being delivered via the AI-generated personalized videos, and took a variety of visual forms. For example, the course introduction and conclusion were “talking head” videos of the instructor, the field trips were in a variety of contexts from UCI’s recycling center to an island on the Great Barrier Reef, and the teaching staff meeting updates were a Zoom recording with the instructor and all the TAs/readers.

The 300 videos (100 topics with 3 variants each) received a total of 17552 views across both quarters. These view counts are based on YouTube statistics; therefore, they may include a small number of views from people outside the course. Anecdotally, though, based on the YouTube analytics charts, the vast majority of the views were during the weeks when the content was assigned. View counts varied dramatically based on when the video was assigned across the quarter. As an example, the first set of videos from week 1 had a total of 909 views across both offerings of the course (534 students), demonstrating that many students likely watched multiple variants of the video (e.g., to see how they were different) or watched the same variant multiple times. Of these views, 388 were views of the Business variant, 276 of the Social/Biological variant, and 245 of the Technology variant. For comparison, the first set of videos in week 5 had a total of 149 views across the two offerings of the course, and the first set of videos in week 9 had a total of only 41 views, demonstrating that fewer than 10% of the course population was watching the videos voluntarily by the end of the course. The drop off from weeks 5 through 9 is likely explained, in part, due to the fact that several of their assignments in weeks 5–8 relied on material from the week 5 videos, whereas the assignments after the week 9 videos did not rely on those videos.

**Fig. 1:**
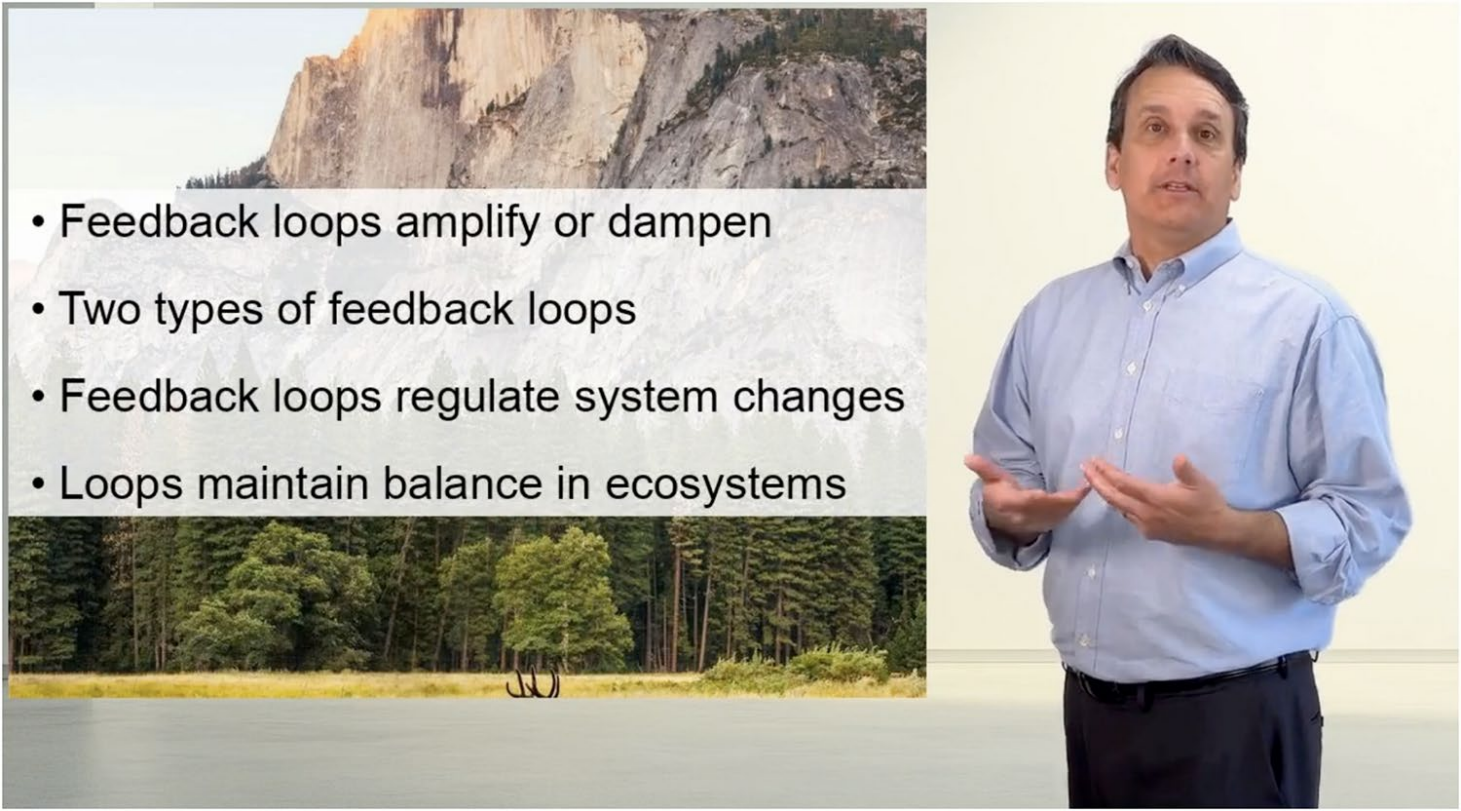
An instance of an AI-generated personalized video.

## Results

### Overview of participants and study design

Participants were drawn from the student populations of two offerings of the large online undergraduate course described above. Of the 534 students in these offerings, 14% were first-year students, 15% sophomores, 12% juniors, 54% seniors, and 5% unspecified. The predominance of seniors likely reflects students finishing the General Education requirements, one of which this course satisfied. The unspecified students were those enrolled from a different UC campus. A total of 493 students (255 in the Spring 2025 offering and 238 in the Fall 2025 offering) completed an end-of-term survey, representing 92.3% of the 534 students enrolled across both offerings of the course. Students provided complete rankings of four educational video types defined by two orthogonal dimensions: personalization (personalized versus non-personalized) and source (human-recorded versus AI-generated). The four video categories were: human-recorded personalized, AI-generated personalized, human-recorded non-personalized, and AI-generated non-personalized. Human-recorded personalized videos were a hypothetical type, since no videos of this type were produced; students ranked this category based on what they imagined such videos would be like. AI-generated personalized videos were the main modality used in the courses under study (see Fig. 1). Human-recorded non-personalized videos are the standard for many online courses; students’ conception of this category likely drew on both the human-recorded videos in this course and their broader prior experience with online lecture videos. We assumed familiarity with this genre, since approximately 3/4 of students who enroll in this course have taken previous online courses and most of the remainder have familiarity with YouTube and other instances of the human-recorded non-personalized genre. This figure is derived from surveys given in two previous offerings of the same course, in which 73% and 74% of students had taken one or more previous online courses. AI-generated non-personalized videos corresponded to the same AI-generated video content used in the course, but without personalization cues, and thus represented a generic instance of AI-generated instructional video for each topic. Importantly, the four conditions differ in how directly students experienced them, a point we return to in the Limitations section.

### Overall preference structure

**Fig. 2:**
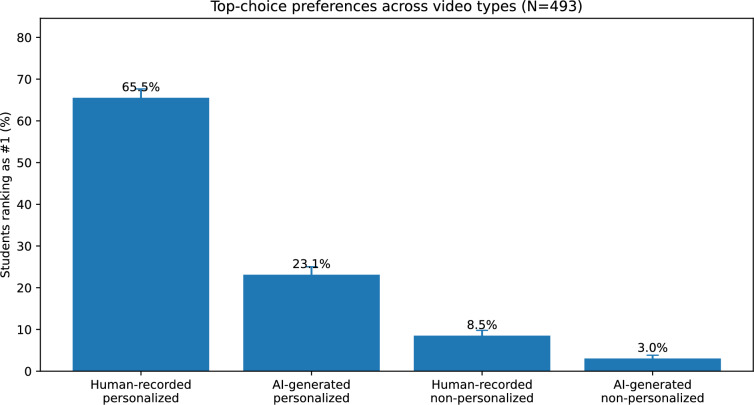
Student top-choice preferences for educational video types. Bars show the percentage of students who ranked each video type as their most preferred option. Data combine two cohorts (Spring and Fall; $N = 493$). Personalized videos (human-recorded or AI-generated) accounted for the vast majority of first-place rankings, whereas non-personalized videos were rarely selected as most preferred.

Across both cohorts, student preferences exhibited a clear and consistent structure. On average, hypothetical human-recorded personalized videos received the highest preference rankings, while AI-generated non-personalized videos received the lowest (see Fig. 2). This ordering was stable across course offerings.

### Effect of personalization

When instructional formats were grouped by personalization (collapsing across human-recorded and AI-generated content), 88.4% of students ranked either human-recorded or AI-generated personalized videos as their top choice. This proportion was highly above chance (two-sided binomial test against 0.5, $p < 0.001$) and was consistent across both cohorts. The same ordering was observed within both human-recorded and AI-generated video categories. Nearly nine out of ten students ranked a personalized video first, indicating a strong and robust preference for instructional content tailored to individual interests.

### Effect of human presence

When instructional formats were grouped by source (collapsing across personalized and non-personalized content), 73.8% of students ranked human-recorded videos as their most preferred option. This preference was also significantly above chance ($p < 0.001$), but smaller in magnitude than the effect of personalization.

Taken together, these results indicate that human presence remains highly valued in educational video, particularly when combined with personalization, and that human-recorded instruction is preferred overall when personalization is held constant. However, when personalization and human presence are placed in direct contrast, students preferred AI-generated personalized videos over non-personalized human-recorded videos by a substantial margin, indicating that personalization can outweigh human presence under realistic instructional tradeoffs.

### Mean rank analysis

Mean rank data reinforced these findings. In the Spring cohort, human-recorded personalized videos had a mean rank of 1.44, followed by AI-generated personalized videos (2.31), human-recorded non-personalized videos (2.69), and AI-generated non-personalized videos (3.56). The Fall cohort showed a nearly identical pattern (1.57, 2.21, 2.69, and 3.52, respectively). Across both cohorts, AI-generated personalized videos received a significantly lower (more preferred) average rank than human-recorded non-personalized videos (mean rank 2.26 vs. 2.69; Wilcoxon signed-rank test, $p < 0.001$). This preference indicates that, at least in the context of this course’s content and this student population, the benefits of personalization outweighed the presence of a human instructor.

### Written feedback

Although we did not conduct a formal qualitative coding of students’ open-ended responses, student comments revealed consistent and interpretable patterns that help contextualize the quantitative results. Students articulated recurring benefits and drawbacks of the AI-generated personalized videos, sometimes acknowledging both within the same response.

On the positive side, many students emphasized the value of personalization and relevance. Personalized examples were frequently described as increasing engagement and helping students see how course concepts connected to their own interests or future goals. One student wrote that the videos “felt unique and personalized to me, which kept me engaged during the course,” adding that it felt as though “the course was catering to me based off of my interests.” Another noted, “I benefited from the tailored facts based on my own career interests... Often in courses, I zone out because I feel the information isn’t tailored to something that will benefit me.”

Students also repeatedly highlighted clarity, consistency, and conciseness as strengths of the AI-generated videos. Compared to prior human-recorded videos the students had experienced, AI videos were often described as more focused and easier to follow. As one student explained, “These AI-generated videos are more direct and concise than traditional instructor-made videos which tend to be longer, sometimes filled with jamble. [sic]” (We assume that “jamble” is a conflation of “jargon” or “jumble” and “ramble”, all terms that describe speech patterns of which professors are sometimes guilty.) Another commented, “AI-generated videos get straight to the point without any unnecessary fluff – a big plus since they don’t waste my time.” Several students noted that the consistent pacing and articulation made the videos easier to understand, particularly when taking notes or following captions. This consistency was an unexpected benefit of the convergence of the 5-paragraph format and the AI rendering for which our production pipeline was optimized. The targeted course enrolls a relatively large population of English Language Learners (ELL students); the standardization, crisp diction, and complete sentences of AI may be a benefit for these and other populations.

At the same time, students articulated clear and recurring concerns about AI-generated instruction. The most common critiques centered on perceived unnaturalness and a lack of human presence. Many students described the videos as monotone, robotic, or unsettling, with several explicitly invoking uncanny valley effects. One student wrote, “There is just an uncanny feeling watching the videos. It doesn’t feel natural,” while another noted, “Uncanny valley really creeped me out. I kept getting goosebumps.” Others emphasized a diminished sense of personal connection, stating that the videos “lacked ‘life’ or feeling as if a professor was actually talking to me” and that they “seemed cold and missing the human charm”.

A smaller number of students raised concerns about trust and authenticity, questioning whether AI-generated content had been adequately verified by a human. For example, one student remarked that the videos felt “untrustworthy, especially since I was not sure if the material generated was verified by a ‘human.”’ This comment was provided despite assurances in week 1 that all material was vetted by the instructor. But students may not have fully internalized that oversight process. A few students also referenced broader ethical concerns, including the environmental impact of AI technologies, particularly in the context of a course focused on sustainability.

## Discussion

This study examined a central tension in contemporary online education: personalization is widely associated with higher engagement, yet AI-generated video is often perceived as less appealing than human-recorded instruction. Across two offerings of a large undergraduate online course, students’ rankings reveal that personalization played a substantial role in shaping preferences. This finding is notable because the study measures perceived value and preference, constructs that are meaningful in their own right as indicators of technology acceptance^15^, not merely as proxies for learning outcomes. In particular, many students preferred AI-generated personalized videos over non-personalized human-recorded videos, indicating that perceived relevance and clarity can outweigh the value students place on a real instructor’s face and voice under realistic instructional tradeoffs. At the same time, human-recorded personalized videos were rated most highly overall, underscoring that human presence remains strongly valued, especially when paired with personalization. Taken together, these results suggest that while human presence continues to matter in educational video, personalization can be the more influential factor when students must choose between personalized and generic instruction.

These results align with decades of evidence that individualized instruction can produce large gains in learning, but has historically been difficult to deliver at scale^5^. In this view, visible human presence functions as a social signal that enhances how instruction feels, which helps explain why AI-generated teaching videos can achieve comparable learning outcomes while still being evaluated less favorably on experiential dimensions^11^. In other words, students can simultaneously: (a) notice that human video feels better and (b) still choose AI when AI delivers more personally relevant content. From this perspective, personalization and social presence operate as partially substitutable signals of instructional care: human delivery enhances affective experience, while personalization enhances relevance, and students appear willing to trade one for the other.

The qualitative feedback in this study supports that interpretation. Students frequently described the AI-generated personalized videos as more concise and focused, echoing prior findings that video design and production choices influence engagement (e.g., shorter and more tightly scripted videos tend to retain attention better)^1^. While brevity is not solely a characteristic of AI-generated content, AI does tend to stick to a script more reliably than a human does. At the same time, students raised familiar concerns about synthetic presenters, including reduced expressiveness and uncanny-valley discomfort^13^. Taken together, the pattern looks less like “AI hype” and more like an ordinary tradeoff: some students will accept weaker social presence in exchange for higher personal relevance. This tradeoff has practical implications for the design of scalable instructional systems: rather than attempting to maximize human likeness in AI presenters, designers may achieve greater impact by prioritizing relevance, concision, and alignment with learner interests, while selectively reintroducing human presence where social connection is pedagogically essential. Note that this is only a relevant tradeoff because the students were told and/or could tell that the videos included AI-generated avatars; as the technology improves, the AI videos may become indistinguishable from, or potentially even superior to, real human video. It is also possible that an uncanniness threshold exists beyond which personalization would no longer compensate for discomfort with synthetic presenters, a question that future work should investigate as avatar fidelity continues to improve. As AI-generated presenters approach perceptual indistinguishability from human presenters, the personalization–presence tradeoff documented here may shift substantially, potentially rendering it moot if learners can no longer detect the difference.

### Limitations and alternative explanations

Several limitations matter for interpreting these results. First, only one of the four ranked conditions (AI-generated personalized videos) was delivered as the primary instructional modality; the “human-recorded personalized” condition was hypothetical rather than experienced, the “AI-generated non-personalized” condition may not have been experienced as a parallel alternative to the course lectures, and the “human-recorded non-personalized” largely relied on students’ experiences with other courses. Rankings therefore mix lived experience with informed imagination.

Relatedly, the experiential asymmetry across conditions introduces the possibility of what might be termed “imagination bias.” When students ranked human-recorded personalized videos, a category they had not experienced, their mental image may have diverged from what such videos would actually look like. This bias could operate in either direction. Students may have envisioned an idealized version: a charismatic instructor delivering perfectly tailored content without the technical imperfections of current AI avatars, a standard that may be difficult or impossible to achieve at scale. Alternatively, students may have imagined something more modest, such as a conventional lecture that merely references their major in passing, underestimating what a dedicated human-recorded personalized effort could achieve. The direction and magnitude of this bias are unknown, and the resulting uncertainty applies to any comparison involving the hypothetical condition. What can be said is that AI-generated personalized videos ranked second overall despite being evaluated against a condition whose true quality is unconstrained by reality; whether that works for or against them depends on assumptions we cannot test with the present data.

The four video conditions also differed on dimensions beyond personalization and source. The personalized AI-generated videos followed a structured five-paragraph script and were typically 3–5 minutes long, whereas the human-recorded videos included field trips, expert interviews, anecdotes, and varied substantially in format and length. (The non-personalized AI videos were more varied in format, including an introduction to AI delivered by the AI avatar and end-of-week summary videos featuring playful personas such as a pirate and a wizard, demonstrating that the AI production pipeline was not inherently constrained to a single format.) These functional differences mean that the comparison between conditions reflects not only personalization and human presence but also differences in production format, conciseness, and content type. This is a characteristic of the field-deployment design rather than a limitation that could be easily removed: the formats under comparison reflect the realistic tradeoffs that instructors face when choosing between scalable AI-generated content and traditional human-recorded instruction. Future work using more tightly controlled experimental designs could isolate the effects of personalization and source from these co-varying production characteristics.

Beyond these design-related caveats, several additional limitations bear on interpretation. Preference rankings are about perceived value rather than demonstrated learning outcomes; prior work suggests these can diverge (e.g., similar learning with different enjoyment)^11^. A companion study is currently underway examining objective learning outcomes across video conditions, using assignment grades as the primary measure. While we do not report those results here to avoid premature conclusions from incomplete data, the combination of preference data (this study) and learning outcome data (forthcoming) will provide a more complete picture of the effects of personalized AI-generated instruction.

Additionally, demand characteristics and social desirability (e.g., students “telling us what we wanted to hear”) may have influenced some responses, particularly in a course context where students knew about the goals of the research being undertaken by the instructional team. A formal qualitative analysis of the open-ended responses, including systematic coding and thematic analysis, is planned as a separate study; the illustrative use of student comments in the present paper is not intended to substitute for such an analysis.

Further, many students had prior exposure to online courses, and for most of them, those courses relied on non-personalized, human-recorded lecture videos. Preferences in our study may therefore partly reflect a contrast effect: students’ baseline expectation of “generic lecture video” could amplify the perceived value of personalization when encountered for the first time. If so, the strong preference for personalized videos may overstate the long-term effect, reflecting novelty rather than a stable preference. Whether personalization retains its appeal after repeated exposure across multiple courses remains an open question.

Finally, this study took place within a single large online course at one research university, fulfilling a general education requirement. The student population was predominantly seniors, the course was fully online, and the subject matter (sustainability and computing) may attract students with particular orientations toward technology. These contextual factors may limit the generalizability of the findings to other course formats (e.g., in-person, hybrid), disciplines, institution types, or student populations. Replication across varied educational settings is needed. Future work should isolate these factors through randomized exposure to parallel conditions and include learning and persistence outcomes in addition to preference.

### Ethics and governance

Scaling personalization to a finer-grained level requires collecting or inferring student attributes (interests, background, prior knowledge), which introduces privacy and governance challenges. It also raises concerns about bias, stereotyping, and uneven quality if personalization pipelines are not audited and instructor-supervised. Recent guidance on the use of generative AI for education emphasizes human-centered governance, transparency about AI use, and protections for learners’ data^22^. A conservative approach, such as was used in the course described in this study, includes the teaching staff driving the generated content, clear communication to students, and minimizing collection of sensitive personal data.

A separate ethical consideration concerns students who object to the use of generative AI on principled grounds, including environmental sustainability, labor practices, data sourcing, or broader concerns about automation and academic integrity. The courses in which we collected the data for this article included one reading^23^ and several videos that focused on aspects of the environmental impact of AI, which may have contributed to student awareness of these concerns. Unlike usability issues (e.g., expressiveness or naturalness), these objections may not be mitigated by technical improvements alone. As AI-mediated instructional systems become more deeply embedded in educational infrastructure, students may experience a diminishing ability to meaningfully opt out, raising questions about consent, autonomy, and moral coercion in learning environments. While the present study did not systematically examine these objections, several students articulated discomfort or resistance to AI-generated instruction for explicitly ethical reasons. Addressing such concerns may require institutional policies that go beyond technical governance, such as providing alternative instructional pathways, clearly articulating pedagogical justifications for AI use, and engaging students in transparent discussion about the tradeoffs involved. Future work should examine how ethical opposition to AI shapes student engagement and whether inclusive design strategies can accommodate principled non-use alongside scalable personalization.

### Conclusion

Taken together, these findings suggest that student perceptions of educational video may be reaching a turning point. While the limitations of AI-generated instruction, such as reduced expressiveness and perceived artificiality, remain salient, their impact appears, for many students, to be outweighed by the personalization benefits that generative AI can provide. In this study, personalized AI-generated videos were often preferred to non-personalized human-recorded lectures, indicating that relevance and contextual fit can now compensate for, and in some cases surpass, the absence of a human presenter.

Human-recorded instruction continues to be valued, particularly for social presence, authenticity, and emotional connection. However, the results suggest that generative AI has crossed a threshold at which its remaining weaknesses are no longer the dominant factor in students’ evaluations of instructional video. Instead, AI’s ability to deliver individualized content may mark the emergence of a new phase in online education: one in which personalization becomes a central design principle rather than a luxury.

Rather than framing the future of education as a choice between AI and instructors, though, these findings may instead point toward a complementary model: human instructors provide expertise, mentorship, and social connection, while AI systems extend their reach by enabling personalized instruction that would otherwise be infeasible. Such a hybrid approach may represent a meaningful evolution in online learning, combining the strengths of both human and machine in ways that better meet the needs of diverse learners.

## Methods

### Participants and context

The study was conducted in a large undergraduate online course at the University of California, Irvine, an introductory course on sustainability and computing that fulfilled a “Science and Technology” general education requirement. Data were collected across two academic quarters: Spring 2025 and Fall 2025. Course enrollment included 534 students across both offerings. End-of-term survey responses were received from 493 students (255 in Spring 2025 and 238 in Fall 2025), yielding a response rate of 92.3%. The study protocol was approved by the Institutional Review Board at the University of California, Irvine (UCI IRB #5377: *AI-Enhanced Personalization for Large-Scale University Education in Sustainability Science*). The need for informed consent was waived due to its classification as exempt research, that is, “[r]esearch, conducted in established or commonly accepted educational settings that specifically involves normal educational practices that are not likely to adversely impact students’ opportunity to learn required educational content or the assessment of educators who provide instruction”^24^. The data used were deidentified and analyzed after the course was completed. All methods were carried out in accordance with relevant guidelines and regulations.

### Survey and ranking procedure

At the conclusion of each quarter, students completed an online survey regarding various aspects of the course. One component of the survey was a ranking task in which students were asked to rank four types of educational videos from most preferred (rank 1) to least preferred (rank 4):

- Human-recorded, non-personalized videos
- Human-recorded, personalized videos
- AI-generated, non-personalized videos
- AI-generated, personalized videos

Students completed the ranking via a drag-and-drop interface.

The survey also included two open-ended questions focused on the personalized AI-generated videos: (1) “What is one specific benefit that you experienced with the personalized AI-generated videos?” and (2) “What is one specific drawback that you experienced with the personalized AI-generated videos?” Students were instructed to write “None” if they did not perceive a benefit or drawback. These open-ended responses are used in this paper to illustrate and contextualize the quantitative ranking data. We did not conduct formal qualitative coding (e.g., thematic analysis with inter-rater reliability) of these responses; a dedicated qualitative analysis with an appropriate methodology is in preparation as a separate study.

### Quantitative analysis

Each participant provided a complete ranking of the four video types. Rankings were aggregated to compute mean rank values for each content type, with lower mean ranks indicating higher average preference. We also calculated first-choice frequencies (the percentage of students assigning rank 1 to each type). Results are reported separately by cohort and combined across cohorts, as patterns were highly consistent between Spring and Fall offerings.

Post-hoc pairwise comparisons were conducted using Wilcoxon signed-rank tests for paired ordinal data, with Bonferroni correction applied for multiple comparisons. Key comparisons of interest included AI-generated personalized videos versus human-recorded non-personalized videos. All reported pairwise differences were statistically significant at $p < 0.001$ after correction.

### Use of AI

Portions of this article were drafted and/or revised using an AI system (ChatGPT-5.2, Claude Opus 4.6) as a writing support tool. All content was reviewed, verified, and approved by the authors. The use of AI tools followed established best practices for ethical, transparent, and responsible scholarly writing^25,26^.

## Data Availability

De-identified ranking data supporting the findings of this study are publicly available at: https://doi.org/10.17605/OSF.IO/TAZCW.
